# Frailty and Intrinsic Capacity: Two Distinct but Related Constructs

**DOI:** 10.3389/fmed.2019.00133

**Published:** 2019-06-18

**Authors:** Giulia Belloni, Matteo Cesari

**Affiliations:** ^1^Specialisation School in Geriatrics, University of Milan, Milan, Italy; ^2^Department of Clinical Sciences and Community Health, University of Milan, Milan, Italy; ^3^Geriatric Unit, Fondazione IRCCS Ca' Granda Ospedale Maggiore Policlinico, Milan, Italy

**Keywords:** older people, healthy aging, person-centered care, comprehensive geriatric assessment, disability, public health

## Abstract

Frailty is a clinical condition characterized by the individual's increased vulnerability to endogenous and exogenous stressors. It is determined by the reduction of homeostatic capacities of the organism and responsible for a marked risk of adverse health outcomes (including functional loss and mortality). Frailty originates from the geriatric background and may pave the way toward a model of care centered on the person, deviating from the traditional and obsolete disease-focused approach. Unfortunately, many controversies have affected the field of frailty over the years and ambiguities have been growing. In particular, the common use of frailty as condition to “exclude” from interventions is a worrisome trend. In fact, the detection of frailty should instead represent the entry point for a more in-depth analysis with the aim of identifying the causes of individual's increased vulnerability and implementing a person-tailored intervention plan. With the aim of promoting a more comprehensive and appropriate assessment of the aging population, the World Health Organization introduced the concept of intrinsic capacity (IC), defined as the composite of all physical and mental capacities that an individual can draw upon during his/her life. Frailty and IC are two constructs stemming from the same need of overcoming traditional medical paradigms that negatively impact on the correct way clinical and research practice should be conducted in older persons. In this article, we describe the similarities and differences between the two constructs, highlighting how geriatric medicine contributed to their development and will be crucial for their further integration in future healthcare models.

## Background

The right to health is applicable to all ages, including the later years of life (1). There is wide variability among terms and meanings used to encapsulate the notion of “aging well,” as healthy aging, successful aging, active aging, positive aging, productive aging (2). In Table 1 are presented the concepts of healthy aging, active aging and successful aging, which are often alternatively used.

**Table 1:** Terms to design “well aging.”

Healthy aging	The process of developing and maintaining the functional ability that enables well-being in older age. Functional ability is about having the capabilities that enable all people to be and do what they have reason to value. This includes a person's ability to meet their basic needs, to learn, grow and make decisions, to be mobile, to build and maintain relationships, and to contribute to society (3). Healthy Ageing replaces the World Health Organization's previous Active ageing: a policy framework developed in 2002. Healthy Ageing, like active ageing, emphasizes the need for action across multiple sectors and enabling older people to remain a resource to their families, communities and economies (4).
Active ageing	The process of optimizing opportunities for health, participation and security to enhance quality of life as people age (3). The key determinants of active ageing are: economic, behavioral, personal, social, health and social services, and the physical environment (5).
Successful aging	It is an evolving concept without an unique consensus definition and operationalization (6, 7). The main constructs considered are physiological constructs, engagement constructs, well-being constructs, personal resources and extrinsic factors (e.g. finances) (8). Some conceptual models of successful aging are: Rowe and Kahn's model of successful aging (9) based on the balance of three components: avoiding disease and disability, high cognitive and physical function and engagement with life. Selective Optimization with Compensation (10) successful aging is regarded as a lifelong process of maximizing gains and minimizing losses by means of three processes: selection, optimization, and compensation. Preventive and Corrective Proactivity (11) emphasize the critical role of proactive behavioral adaptations in ameliorating the adverse effects of stressors. Phelan at al. (12) definition of successful aging is multidimensional, encompassing physical, functional, psychological, and social health. Pruchno et al. (13) define successful aging as a multidimensional construct having both objective and subjective dimensions. Bowling and Dieppe (14) regard the successful aging as an ideal state to be aimed for, and the concept itself should be placed on a continuum of achievement rather than subject to simplistic normative assessments of success or failure.

The promotion of healthy aging, regarded not as the absence of diseases but as the process for fostering and maintaining the individual's functional ability, has been set as a priority by the World Health Organization (1). To achieve this goal it is necessary to change our healthcare systems which are traditionally centered on the concept of diseases in favor of a new paradigm giving value to the person's functions and values (3, 15, 16).

Under this perspective, both frailty and intrinsic capacity may help clinicians at understanding the complex health needs and priorities of older people as well as trigger tailored actions to promote healthy aging (17).

## Frailty Definition and Assessment

Frailty is defined as an age-related medical syndrome, caused by multiple causes and contributors negatively affecting the homeostatic reserves of the individual. The resulting accentuated vulnerability predisposes the person to high risk of negative outcomes (e.g., geriatric syndromes, hospitalization, institutionalization, disability and death) (18). Although a theoretical definition of frailty is almost universally agreed upon (19), there is a lack of corresponding consensus about the wide range of instruments which are available for use in clinical practice (20).

The phenotypic model of frailty proposed by Fried and colleagues (21) and the one based on the age-related accumulation of deficits published by Rockwood and Mitnitski (22) are widely recognized as the two main schools of thought in the field. The frailty phenotype is focused at evaluating the physical domain of the individual by considering five pre-defined signs and symptoms. It is conceived as a categorical variable, discriminate frail from robust and pre-frail individuals. By providing a clear conceptual framework, it has largely contributed toward raising awareness of frailty as an often neglected clinical manifestation of the older person, largely determined by sarcopenia and muscle exhaustion. In contrast, the Frailty Index is designed as a continuous variable computed from the results of a comprehensive geriatric assessment (CGA). Its quantitative nature is based on the assumption that (1) health deficits (i.e., signs, symptoms, diseases, disabilities and laboratory abnormalities) tend to accumulate with aging, and (2) the higher number of information generates a more robust instrument (23). It is thus evident that the instrument is not based on a predefined set of variables (that is, it cannot be computed without adequately knowing the person).

Besides of these two main instruments, frailty can be measured with a huge variety of instruments. It is noteworthy that even physical performance tests (e.g., usual gait speed, Short Physical Performance Battery, Timed “Up & Go”) can also be used for measuring the frailty status of the individual (24, 25). In fact, although they were designed for objectively assessing a specific capacity of the organism, they are still able to adequately capture the underlying biological age of the individual and thus support clinical decisions in the planification of an adapted model of care. In this context, it is important to consider how the choice of a screening tool should not be limited to rigid conditions imposed based on a specific tool. In fact, the choice of frailty instrument should rather be driven by the purpose of the frailty identification. Thus, the instrument should preferentially be simple to use, validated and provide a language to appropriately guide goal setting and care planning such that identification of frailty is able to meaningfully impact the management of the individual in a contextual and appropriate way (26, 27).

## Benefits of Assessing Frailty in Clinical Practice

The assessment of frailty in clinical practice is useful insofar as its detection is able to inform and modify the decisional algorithm. Screening a condition as an endpoint unto itself renders the initiative meaningless and can even be detrimental from the ethical and economic standpoint. The identification of frailty in the clinical setting should lead to the analysis of the causes of and contributors to the increased vulnerability of the individual (i.e., comprehensive geriatric assessment). As such, frailty may become the entry point to a model of adapted care for individuals defined as at increased risk of negative outcomes on the basis of a non-traditional paradigm (e.g., diseases, old age).

If left untreated, frailty may initiate a vicious circle characterized by the worsening of the individual's health status and disabling cascade (28). However, frailty might be potentially reversible; therefore, its detection should lead to the implementation of preventive strategies for correcting abnormal deviations from the normal trajectory of aging.

In this scenario, frailty may assume the meaning/value of a marker of biological aging. It will then possible to corroborate clinical decisions with objective data going beyond traditional constructs and nesting the concept of function and biological reserves in the assessment of the aging person. It implies the possibility of proceeding toward the personalization of care, accepting that chronological age does not adequately correspond with the individual's biology (especially in our aging world where the older population is extremely heterogeneous) (29–31).

Nowadays, the concept of frailty is increasingly used (even outside the perimeter of geriatric medicine) as a measure of an individual's risk profile. Patients are today often stratified on the basis of frailty, which consequently becomes a sort of triage method in the planning of a certain intervention. It is important to clarify that the frailty was not conceived by geriatricians as a condition for “excluding” from interventions. Rather, it was designed for the exact opposite purpose, that is include and extend interventions to persons who may be traditionally excluded based upon tenuous assumptions of old age. The detection of frailty should indeed represent the entry point for a more in-depth analysis of the individual (i.e., the comprehensive geriatric assessment) with the aim of identifying the causes of his/her increased vulnerability, implementing a person-tailored intervention plan. In other words, frailty should be considered as a condition to rule-in rather than rule-out, to include rather than exclude, a matter for working more rather than a STOP sign.

## Intrinsic Capacity

The concept of intrinsic capacity (IC) was introduced by the World Health Organization in 2015 in order to create a multidimensional indicator related to individual's functional status whose follow up over time may be useful to reach the healthy aging. In the World Report on Aging and Health, IC was presented as a new model for capturing in a holistic way the individual's functions and capacities adopting a life-course approach. IC is defined as the composite of all the physical and mental capacities of the person (3), and represents the amount of resources one can tap into during his life. By interacting with the surrounding environment, IC largely defines the individual's functional ability (i.e., what the person can aspire to do for giving value to his/her abilities and positively act for the society).

IC is a dynamic construct and its trajectory over time may inform clinical and public health actions as soon as its monitoring is contextualized at the individual or population level, respectively (32). In fact, by following its trajectory over time, the clinician may identify deviations from normality before the onset of clinical manifestations (and thus preventively act in the maintaining of healthy aging), or evaluate the effectiveness of interventions. At the same time, public health authorities may detect regions or populations at special need of attention when presenting critical signs of poor capacities and declines (33).

On the other hand, considering the present lack of developed and validated tools to measure the IC, future practical implementation of IC that enables quantification of longitudinal changes will enable the field to define a clinically relevant difference in term of one's IC.

The IC model is also based on the rationale that the individual's capacities tend to fade with aging. At the same time, the environmental barriers become more burdening, increasing the gap between what the person may do and what in reality does. The solution proposed by the WHO model is to increase IC and/or reduce the environmental barriers in order to allow older persons to (1) do what they have reason to value, and (2) make them again active and functional in the society where they live.

## Intrinsic Capacity Assessment

To date, the IC model is still at its infancy and for now, a largely theoretical construct. The WHO has, for now, only officially presented the domains theoretically defining IC. Thanks to a review of the literature, five domains/functions, which are deemed critical for adequately and comprehensively capturing the IC, have been selected (34). These domains are the locomotion, vitality, sensory (in particular, vision and hearing), cognition, and psychological ones. These domains influence each other and are in turn all influenced by environmental factors. More recent discussions have focused their attention on the concept of vitality, which has developed over time from one of the five domains defined by the energy metabolic capacity of the person to a kind of background biological reserve from which the other four domains stem and act.

A process for translating theory into practice is ongoing. In particular, efforts are devoted to provide instruments to clinicians and researchers able to capture and render objective IC in the next future. The World Health Organization is currently coordinating the activities of a Clinical Consortium for Healthy Aging, which is promoting the clinical implementation of the IC model and the integration of care services for older persons (35). WHO will shortly provide the international validation of the operational definition of IC and the tools/instruments for measuring the five domains and objectifying one's functions.

The concept of IC is closely related with the integration of services. This link implies the fact that, being IC not monodimensional, multiple aspects have to be measured and considered for the correct planning of interventions (36). At the same time, these latter will likely require to be multicomponent, multidisciplinary, and coordinated. It is, in fact, evident that a complex matter may find solution only through the implementation of a comprehensive and adequately targeted plan of action. The interventions to bring the aging individual back to an acceptable level of functional ability may result in the increase of IC (i.e., improving the individual's reserves/functions) and/or reducing the environmental barriers. As is well-known from geriatric medicine literature, the interventions will thus require an extension of the evaluation to the context where the individual lives and where, very often, solutions to health deficits can be found. To date, given the still theoretical nature of IC construct, the WHO has not yet provided specific indications on how to operationalize the environment factors in the equations between intrinsic capacity and functional ability.

## Frailty and Intrinsic Capacity

Frailty and IC present many similarities as well as some peculiar/characterizing points (Table 2). Under certain aspects, IC might be considered as a sort of evolution of frailty. The development of frailty into something “new” is motivated by multiple reasons as: (1) the need of disseminating the comprehensive approach to the older person beyond the perimeter of geriatric medicine (even in countries where geriatricians are relatively absent), (2) the necessity to provide a positive connotation to the aging phenomenon (thus focusing on functions rather than on deficits), (3) the importance of working on trajectories instead of focusing on arguable cross-sectional cut-points, and (4) the attempt to anticipate as much as possible the self-empowerment of the individual for his/her health status (thus supporting preventive strategies in the community).

**Table 2:** Comparison between frailty and intrinsic capacity.

	**Frailty**	**Intrinsic capacity (IC)**
Definition	Progressive decline of physiological systems conferring increased vulnerability to stressors and exposing to the risk of adverse health outcomes	Composite of all mental and physical capacities
When	Geriatric condition	After the age corresponding to the median of the local life expectancy at birth
Time dimension	Cross-sectional assessment	Longitudinal assessment for tracking trajectories
Characteristics	Defined by deficits and abnormalities	Defined by reserves and residual capacities
Original purpose	Developed for addressing the unmet clinical needs of the older person	Developed to inform about public health strategies in the promotion of healthy ageing
Interventions	Comprehensive geriatric assessment, possibly within a network of integrated care	Comprehensive intervention based on integration of care and social services

It is noteworthy that both frailty and IC are based on the assumption that the aging individual can be adequately assessed and managed only if comprehensively evaluated and followed in a novel healthcare model based on integration and mutidisciplinarity of services.

Both frailty and IC are focused at promoting the development of person-centered care plans (ability in detecting one's impairments, needs and preferences) and lead to tailored care/healthy strategies to reverse, slow or arrest the losses. Although both frailty and IC are dynamic entities, frailty is mainly used in cross sectional assessment and IC may be used in a longitudinal approach over the time. This last approach has the benefit of tracing trajectories that can inform when take action with the aim of reversing the trend and inform about effectiveness of the interventions implemented or the variation in one's needs.

Apparently, IC and frailty might represent the two faces of the same coin. One is presenting the reserves of the individual, the other the deficits accumulated with aging. In reality, IC should not be seen as the mere opposite of frailty. We believe the two concepts might be complementary. In particular, frailty may represent a state of extreme vulnerability to stressors defined by a clinically relevant reduction of IC (or functional reserves). As such, the monitoring of IC (or the individual's functional reserves) may support the detection of the person's fragilization. At the same time, the measurement of IC in frail individuals may provide additional information to build up the personalized care program centered on the person's values and priorities (37).

## Conclusions

The aging of the populations is a global phenomenon, and not exclusive of high-income countries. Surely, priority and resources may change from country to country. By developing the novel model of IC, the World Health Organization is taking as background the evidence built on frailty by geriatricians over decades, and develop it into a novel model nested in the framework of healthy aging. By doing so, it tries to overcome some of the known weaknesses of the frailty concept, in particular the stigma today applied to individuals experiencing the age-related disabling cascade. The adoption of IC (especially at public health level) should be seen as an opportunity for better disseminate the geriatric culture in our healthcare systems, frequently lacking of the necessary attention to the specific needs of older persons.

## Data Availability

No datasets were generated or analyzed for this study.

## Author Contributions

All authors listed have made a substantial, direct and intellectual contribution to the work, and approved it for publication.

### Conflict of Interest Statement

The authors declare that the research was conducted in the absence of any commercial or financial relationships that could be construed as a potential conflict of interest.
